# Constructing a cross-tissue human knee single-cell atlas identified osteoarthritis reduces regenerative tissue stem cells while increasing inflammatory pain macrophages

**DOI:** 10.21203/rs.3.rs-6247502/v1

**Published:** 2025-05-05

**Authors:** Rajnikant Raut, Amit Chakraborty, Tuhina Neogi, Michael Albro, Brian Snyder, Thomas Schaer, Chao Zhang, Mark Grinstaff, Manish Bais

**Affiliations:** Boston University, Translational Dental Medicine; Boston University; Boston University; Boston University; Center for Advanced Orthopaedic Studies, Beth Israel Deaconess Medical Center and Harvard Medical School; University of Pennsylvania School of Veterinary Medicine; Department of Medicine Section of Computational Biomedicine, Boston University Chobanian and Avedisian School of Medicine, Boston MA 02118; Boston University; Boston University

**Keywords:** Knee OA, atlas, tissue stem cells, macrophage, pain, SDF1, MMP13

## Abstract

Osteoarthritis (OA) affects the entire knee joint; however, cross-tissue molecular mechanisms are poorly understood due to a lack of comprehensive, integrated analysis. We constructed the first comprehensive single-cell RNA sequencing knee OA atlas from articular cartilage, meniscus, synovium, and subchondral bone which showed active communication between them. Healthy synovium and meniscus contain the largest populations of tissue stem cells (TSCs) and immune cells that are altered in OA. The regenerative TSCs expressing SDF1, SOX9, CD146, PDGFRB, and CD105 decrease during OA, whereas osteogenic TSCs expressing osteogenic differentiation-related factor NT5E (CD73) are increased. In OA, the balance between regenerative and osteogenic TSCs shifts in the OA state with an increased number of osteogenic TSCs. We also report an increased level of quadruple-positive inflammatory (IL1B-IL6-NOS2-TNF) and pain marker (P2RX7) specific macrophages in OA. Fibroblasts are enriched in OA-synovium and may contribute to fibrosis. Importantly, OA cartilage contains unique MMP13-producing detrimental chondrocytes along with RUNX2-producing chondrocytes that worsen OA pathophysiology. This atlas provides a novel avenue for potential therapeutic applications in human knee OA and other musculoskeletal diseases and injuries, targeting synovium and meniscus to intervene in OA-specific molecular and cellular alterations.

## Introduction

Osteoarthritis (OA) vexes over 520 million adults worldwide, manifesting clinically as pain, swelling and decreased joint mobility with associated treatment costs (direct and indirect) approaching $486.4 billion annually. Osteoarthritis (OA) is a prevalent and debilitating joint disease characterized by the progressive degeneration of cartilage, synovium, bone, and meniscus, leading to chronic pain and impaired mobility. The pathophysiology of OA has been related to various biochemical ^1–4^ , biomechanical ^5–7^ , metabolic ^8^ , and gene-regulated ^9^ mechanisms occurring over an extended timeline. Despite its widespread impact, the cellular and molecular mechanisms underlying human clinical OA remain incompletely understood due to the lack of cross-tissue OA atlas. Thus, we constructed the first comprehensive single-cell atlas of the human knee affected by osteoarthritis. By analyzing cartilage, synovium, subchondral bone, and meniscus tissues, we aim to elucidate the distinct cellular populations and their roles in OA pathogenesis. Our investigation includes stem cells, macrophages, and other immune cells, highlighting their contributions to inflammation and tissue remodeling.

Dysregulated balance of tissue degeneration and regeneration in the joint leads to OA pathology, which are governed by tissue stem cells (TSCs). TSCs perform specific functions, and various types of TSCs are enriched in the synovium, meniscus, and cartilage. Studies have identified numerous chondroprogenitor and stem cell markers expressed in TSCs^10–12^. Stem cells differentiate into chondrocytes during OA^13^ and have the potential for cartilage repair^14^. The distinction between various TSCs can help to evaluate the balance of a specific pool. TSCs expressing regenerative factors can be categorized as regenerative TSCs. TSCs expressing factors related to osteogenic differentiation, inflammation, and OA are osteogenic TSCs. TSC pool could be dysregulated with OA, injury and inflammation.

Mechanical overloading or biomechanical changes^15^ that propagate to structural tissue failure (fissuring/fibrillation^16^), tissue swelling^17^, compositional loss of GAG^18^, and derangement of the COLII network^19^. Mechanical injury also incites an inflammatory response and elevated cytokines in OA synovial fluid (e.g., IL-1B, TNF-a).^20,21^. Depletion of cartilage GAG diminishes its rheological and tribological properties^22^, independently contributing to the propagation of OA pathophysiology^23,24^. Joint kinematic dysfunction due to trauma, obesity, or life-long use is one of the main risk factors for OA^25^. The resulting increased mechanical stress causes injury to the principal load-bearing tissues of the knee joint, meniscus, and cartilage^26^ as well as to the synovium and subchondral bone. Damage to even one of these tissues leads to a cascade of alterations in the other tissues, emphasizing the interrelated nature of joint tissue degeneration in OA. All knee joint tissues, including the cartilage, menisci, synovium, and subchondral bone, are affected by OA^27^. Despite this inter-tissue dependence, studies have typically focused on individual tissue types such as cartilage^28^, cartilage and meniscus^29^, synovium and infrapatellar fat pad^30^, and subchondral bone^31^, mainly omitting the underlying cross-tissue mechanisms. Recent developments in single-cell RNA sequencing (scRNA-seq) analysis and genomic technologies have provided insights into the crosstalk between cells in tissues or nearby tissues. As OA is an entire joint disease, analysis of knee joint tissues, including cartilage, meniscus, synovium, and subchondral bone, and their interaction at the single-cell level is of interest and may provide a mechanistic understanding of knee OA development.

In this study, we constructed an atlas of human knee single-cell transcriptomic profiles and identified changes in specific stem cells, macrophages and detrimental chondrocytes. We present a scRNA-seq atlas of 53 human knee joint samples to evaluate the trajectory of cartilage, meniscus, synovium, and subchondral bone cells. Integrated analysis identified OA-specific cell-type clusters as well as inter-relationships between different tissues. The analysis included: 1) The effect and balance of TSCs during healthy and OA conditions; 2) Enrichment of MMP13 or catabolic factor expressing chondrocytes, proinflammatory macrophages expressing quadruple positive markers (IL1B-IL6-NOS2-TNF+), and pain markers such as P2RX7; and 3) identification of co-relative mechanisms for future translation. This knee joint scRNA-seq atlas provides a comprehensive analysis of tissue-specific factors, recognizing crosstalk between the various cells in adjacent synovial tissues that could be targets for therapeutic interventions to mitigate OA. Through this atlas, we identify key cellular subsets and molecular pathways involved in OA, offering potential targets for therapeutic intervention. This work represents a significant step forward in understanding the complex cellular landscape of OA and paves the way for future research and treatment strategies.

## Material and methods

### Human healthy and OA knee specimens.

**Figure 1:**
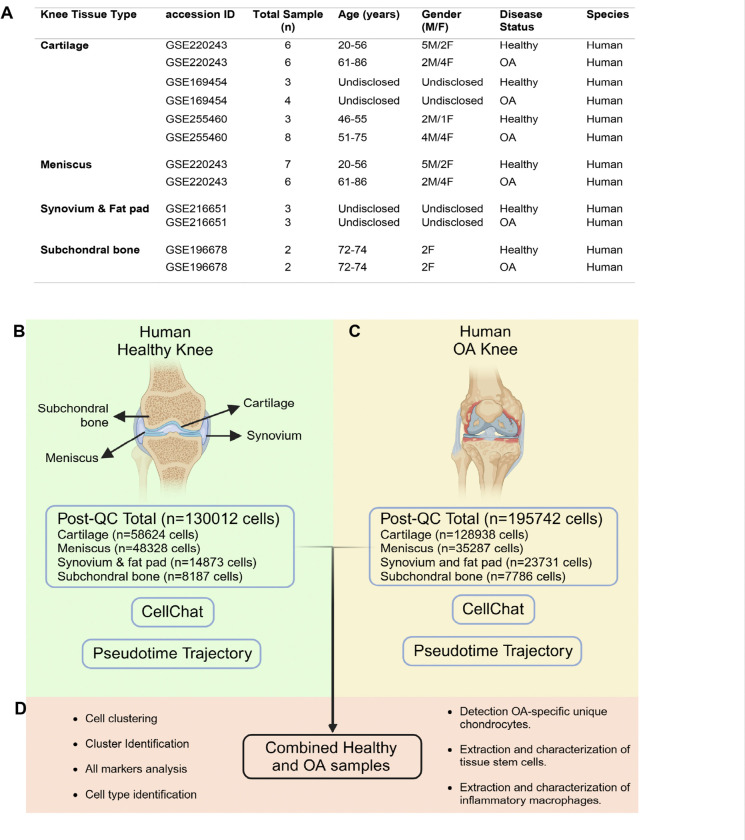
Sample characteristics and study overview. A) Characteristic details of scRNA-seq samples used in this study. B) Anatomy of the healthy human knee and the number of cells retained post-QC using standard Seurat and doubletFinder. C) Anatomy of OA knee and the number of cells retained post-QC. D) Overview of healthy and OA sample integration followed by downstream analyses.

With prior consent from the patients, samples of healthy and OA patients for cartilage, meniscus, synovium, infrapatellar fat pad (SynoFP), and subchondral bone (SCB) were collected from the NCBI Gene Expression Omnibus (GEO) database (please also see Methods and Fig. 1a). The sample description and corresponding accession IDs show specific characteristics that include the entire knee joint tissue. Here, GEO Series (GSE) identifies individual accession ID under which the raw sequencing data files are publicly available (Fig. 1a).

The samples with accession ID GSE220243: Healthy Samples: Matching healthy cartilage (n = 6) and meniscus (n = 7) samples were obtained from the same knee, and only knees with grades 0 or 1 were considered (Graded macroscopically using Outerbridge System). The cartilage samples in their study were collected from the weight-bearing medial femoral condyle, whereas the whole meniscus was collected. OA samples: Corresponding OA samples for cartilage samples (n = 6; grade 4) were collected from medial femoral osteochondral slabs, and meniscus samples (n = 6) were also collected, excluding the ones with large calcium deposits^29^.

The samples with accession ID GSE169454: Non-Arthritic Samples: non-arthritic femoral condyle cartilage specimens (n = 3) were collected from fresh osteochondral allografts discarded during donor plug harvesting during surgical implantation^32^. OA Cartilage Samples: OA cartilage samples (n = 4) were extracted from human patients undergoing total knee joint replacement surgery for OA with prior consent from patients,

The samples with accession ID GSE255460: Non-OA Controls: Samples were collected from three non-OA controls who underwent amputation with no prior history of joint injury or disease.

OA Cartilage Samples: Cartilage samples were collected from eight patients with OA who underwent knee arthroplasty surgery^33^.

The samples with accession ID GSE216651: OA SynoFP: Three OA SynoFP samples were obtained from individuals with end-stage knee OA who underwent total knee replacement surgery.

Non-OA SynoFP: Non-OA SynoFP biopsies were obtained from patients who underwent excision of bone tumors surrounding the knee joint or from organ transplant donors^30^.

The samples with accession ID GSE196678 were SCB samples isolated from the medial and lateral tibial plateaus of two patients with OA undergoing knee replacement surgery. These samples were divided into two groups: control (n = 2) from the lateral tibial plateau and OA (n = 2) from the medial side^31^.

Briefly, all healthy samples from the cartilage, meniscus, SynoFP, and SCB were processed individually for quality control and doublets and integrated using the harmony integration method in Seurat. Similarly, all OA samples were processed and subjected to CellChat^34^ and pseudotime trajectory analysis (Fig. 1b, c). Finally, to perform comparative analysis, both preprocessed healthy and OA samples were integrated and subjected to clustering, cell identity, and cell type proportion analysis (Fig. 1d). This was followed by the extraction of macrophages and TSCs for downstream analyses. As shown in Fig. 1b and 1c, 130012 cells from healthy and 195742 cells from OA were retained after quality control and preprocessing. The total number of cells retained from individual tissue types and analyzed were included (Fig. 1b, c).

### Quality control and preprocessing.

Individual files of Healthy and OA patients single-cell RNA sequencing (scRNA-seq) for knee joint cartilage, meniscus, synovium, infrapatellar fat pad (SynoFP), and subchondral bone were downloaded from the NCBI GEO and processed independently. The zipped files contained a count matrix, features (genes), and barcode files, which were imported into R studio and converted to a Seurat object using the *CreateSeuratObject* function in the Seurat R package (v5.1.0)^35^ with no initial cutoffs. Further details about the sample characterization and categorization are described above.

To filter out low-quality cells, we eliminated cells with less than 3,000 RNA counts, 400 genes (features) detected, and more than 10% reads coming from mitochondrial genes. The retained high-quality cells were further normalized using the default *LogNormalize* method and a scale factor of 10,000 within *NormalizeData* function of Seurat. Clustering was performed with 20 Principal Components (PCs) at a resolution of 0.5, using 2,000 variable features found using *FindVariableFeatures* with the “vst” selection method. The criteria for selecting PCs were defined using elbow plots. Next, *DoubletFinder* R package (v2.0.4) was used to eliminate doublet cells^36^. After quality control and preprocessing 130012 cells from healthy and 195742 cells from OA cells were retained.

### Data Integration and cluster identification.

All individually processed files were then combined into a single Seurat object. Combined files were subjected to data integration using *HarmonyIntegration*^37^ method within *IntegrateLayers* function of Seurat^38^. For downstream processing, 20 PCs were used for harmony clustering at a 0.5 resolution. Healthy samples were combined to understand their cellular and molecular landscapes. The OA samples were combined to understand the cellular and molecular landscapes. Both Healthy and OA samples were integrated to identify common and unique mechanisms.

### Differential marker analysis and cell type identification.

The *JoinLayers* function was used to merge all integrated layers. We performed differential expression analysis using *FindAllMarkers* function to identify the differentially expressed marker genes in each harmony cluster. The Wilcoxon rank-sum test (Wilcox) was used to perform the analysis with *logfc.threshold* of 0.25, singleR (v2.8.0) and celldex (v1.16.0) R packages were used to identify cell types. *HumanPrimaryCellAtlasData* from celldex was considered for this study because it includes almost all types of cells, including stem cells and immune cells^39,40^. TSCs and Macrophages were extracted using *a subset* function of base R for downstream analyses.

### CellChat analysis.

CellChat (v2.1.2) was run on the RNA data slot and separately on the healthy and OA samples.^34,41^. Overexpressed genes were identified using default parameters of *identifyOverExpressedGenes* function. Communication probabilities were computed using trim = 0.1, type = triMean, and other default parameters of *computeCommunProb* function. Communications were filtered to include those with at least 100 cells. The two cell-chat objects were merged to compare their interaction occurrences and strengths. *ComputeCommunProbPathway* function detected actively communicating signaling pathways. Data were visualized in a chord diagram, heatmaps, and scatter plots using the corresponding functional commands of the CellChat package.

### Pseudotime trajectory analysis.

Pseudo-trajectories of chondrocytes and TSCs were constructed using Monocle3^42^. The Seurat object was converted to the monocle3 cell_data_set object using *as.cell_data_set* function and preprocessed, and 20 PCs were retained using the PCA method. Dimensionality reduction was performed using the UMAP method and was clustered using the cluster_ cell function. The *learn_graph* function was used to learn and build the trajectory. O*rder_cells* and *pseudotime* functions were used to order the cells at the pseudotime. Root nodes were selected from the TSCs cluster, considering chondroprogenitor cells as part of the TSCs.

### Data Visualization

Uniform Manifold Approximation and Projection (UMAP) was used to visualize single-cell data. Violine plots, heatmaps, feature plots, and bar plots were generated using *vlnplot* (Seurat), *netAnalysis_signalingRole_heatmap* (CellChat), *Featureplot* (Seurat), and *ggplot2* functions, respectively.

## Results

Cellular and molecular landscape of healthy human knee at single-cell resolution.

**Figure 2:**
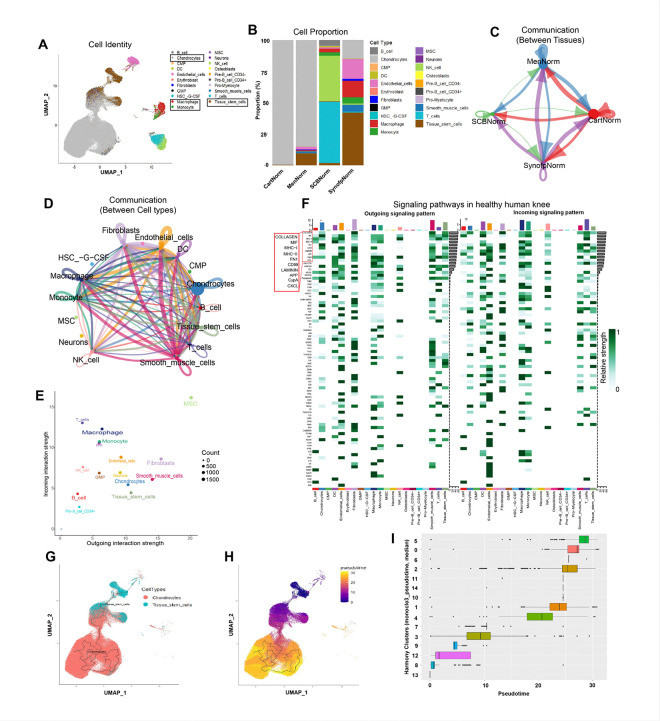
Cellular and molecular landscape of a healthy human knee. A) UMAP representation of total cell types in healthy human knee identified by SingleR. B) Proportion of each cell type in a healthy human knee; here, the chondrocytes predominantly resided in cartilage and meniscus, TSCs in SynoFP and meniscus, and immune cells in SynoFP, subchondral bone, and meniscus. C) Chord diagram showing communication between all four tissue types. D) Chord diagram showing communications between different cell types. E) Scatter plot showing outgoing and incoming interaction strength for different cell types; cell types with molecularly significant differences are shown in the broad letter. F) Heatmap representation of active signaling pathways in the healthy human knee with outgoing and incoming signaling patterns; here, the top signaling pathways are highlighted in the red rectangle. G) UMAP highlighted the location of chondrocytes and TSCs for pseudotime trajectory analysis. H) UMAP visualization of pseudotime trajectory analysis; the root node was selected from the TSCs cluster. I) Horizontal box plot showing pseudotime for individual harmony clusters.

To delineate the cellular and molecular landscapes of the human knee joint and understand the contribution of different tissues to normal function, scRNA-seq samples were integrated. Healthy cartilage, meniscus, SynoFP, and SCB were harmony-integrated, clustered, and subjected to cell identity analysis using the H*umanPrimaryCellAtlas* database in c*elldex* using *SingleR* R packages (Fig. 2a; Fig. S1a, b). UMAP shows all the identified cell types, and Table S1 shows the proportion of each cell type (Fig. 2a). Indeed, the chondrocyte population was dominant and contributed ~ 99.6% of the healthy cartilage samples, whereas the TSCs population contributed only ~ 0.4% of the total cell population (Fig. 2b; Fig. S1b). The chondrocyte population was also enriched in the meniscus, contributing ~ 85.3%, along with ~ 9.5% of TSCs and a small proportion of endothelial cells (EC) (~ 2.7%), smooth muscle cells (SMC) (~ 1.3%), fibroblasts (~ 0.5%), and immune cells (including ~ 0.7% macrophages). However, the proportion of chondrocytes was low in the SynoFP group (approximately 15%) and nearly absent in the SCB group (0.3%).

Interestingly, the TSCs pool was significantly higher in SynoFP, contributing to approximately 42.2% of the total cell population, along with ~ 15.7% ECs, ~ 13.1% macrophages, ~ 5% monocytes, ~ 5.4% SMCs, ~ 1.5% synovial fibroblasts, and a relatively small proportion of other immune cell populations. In SCB, T cells (~ 48.9%) and natural killer (NK) cells (~ 36.1%) together contribute nearly 85% of the total cell population, along with a small proportion of macrophages (2.8%), monocytes (2.7%), TSCs (1.7%), and other immune cell populations. Overall, this data suggests that chondrocytes are predominant in the cartilage and meniscus, TSCs largely reside in SynoFP and the meniscus, and SynoFP and SCB are major reservoirs for the immune cells in healthy human knees.

To identify signaling and communication, CellChat analysis was performed which showed the active communication between all four tissues. The cartilage was a major sender and receiver of signals, followed by the meniscus and SynoFP. SCB was mainly a receiver of signals from the remaining three tissues (Fig. 2c). Analysis of cell types showed MSCs, fibroblasts, SMCs, chondrocytes, and TSCs were the major senders of the signals, whereas MSCs, macrophages, monocytes, dendritic cells (DCs), and T-cells were the major receivers of the signals (Fig. 2d, e). Approximately 85 signaling pathways actively send and receive signals. The most active top 10 signaling pathways are COLLAGEN, MIF, MHC-I, MHC-II, FN1, CD99, LAMININ, APP, CypA, and CXCL (Fig. 2f, Fig. S1d).

Pseudotime trajectory analysis which determines the origin and terminal differentiation state of chondrocytes ^26^, was performed by extracting TSCs and chondrocytes from the total cell population (Fig. 2g). Using TSCs clusters as a root node, we performed the analysis and visualized on the UMAP (Fig. 2h). All four tissues contributed to TSCs and chondrocyte populations; however, the contribution of SCB was negligible (Fig. S1e). Cluster 8 had the lowest Pseudotime since it mostly consists of TSCs and is considered a root node, whereas Cluster 5 and Cluster 0 had the highest Pseudotime, suggesting the terminally differentiated chondrocyte population, primarily residing in the cartilage (Fig. 2i; Fig. S1b, e).

Cellular and molecular landscape of the human knee during OA at single-cell resolution.

**Figure 3:**
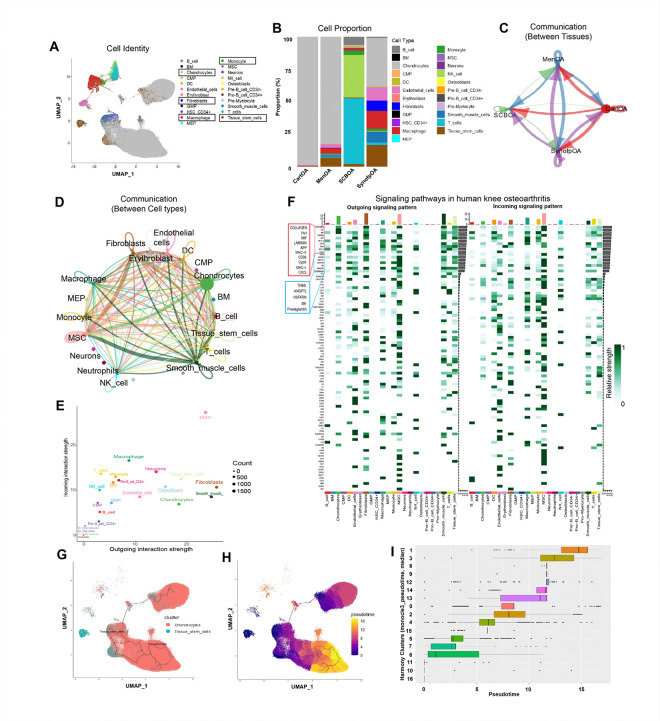
Cellular and molecular landscape during human knee OA. A) UMAP representation of total cell types during OA condition identified by SingleR. B) Proportion of each cell type in the human knee with OA; here, the chondrocytes predominantly resided in cartilage and meniscus, TSCs in SynoFP and meniscus, and immune cells in SynoFP, subchondral bone and meniscus. C) Chord diagram showing communication between all four tissue types. D) Chord diagram showing communications between different cell types. E) Scatter plot showing outgoing and incoming interaction strength for different cell types; cell types with molecularly significant differences are shown in the broad letter. F) Heatmap representation of active signaling pathways in the healthy human knee with outgoing and incoming signaling pattern; the top signaling pathways are highlighted in the red rectangle, whereas OA-specific active signaling pathways are highlighted with the blue rectangle. G) UMAP highlighted the location of chondrocytes and TSCs for pseudotime trajectory analysis. H) UMAP visualization of pseudotime trajectory analysis; here, the root node was selected from the TSCs cluster. I) Horizontal box plot showing pseudotime for individual harmony clusters.

To evaluate the changes in cellular homeostasis during OA, samples were subjected to clustering and cell type identification (Fig. 3a; Fig. S2a, b). Table S2 lists the proportion of each cell type. OA-cartilage has more than ~ 99% chondrocytes population, ~ 0.1% TSCs, 0.4% macrophages and 0.1% monocytes (Fig. 3b; Fig. S2c). In the OA-meniscus, the population was ~ 82.6% chondrocytes, ~ 6.8% TSCs, ~ 3.9% macrophages, ~ 2.8% ECs, ~ 1.2% SMCs, ~ 0.8% monocytes, and 0.3% fibroblasts. OA-synovium had ~ 37.5% chondrocytes and ~ 16.4% TSCs. Interestingly, synovium had a higher number of macrophages (~ 13.7%), fibroblasts (~ 7.7%) and monocytes (~ 1.8%), suggesting increased fibrosis and inflammation in knee OA. In SCB, NK cells (~ 32.6%) and T cells (~ 50.6%) contributed to ~ 83.2% of the total cell population, along with a small proportion of TSCs (~ 2%), macrophages (~ 1.3%), monocytes (~ 3.3%), chondrocytes (~ 1%), and other immune cell populations. Overall, chondrocyte population significantly increased in OA-synovium. In contrast, the TSCs were reduced in OA synovium and meniscus. Macrophage infiltration was increased in the meniscus and synovium. Monocyte infiltration was elevated whereas ECs were reduced in synovium.

CellChat analysis showed OA-cartilage was a major sender and receiver of signals, followed by the meniscus and synovium, whereas SCB was mainly a receiver of signals from the remaining three tissues (Fig. 3c). Unlike healthy tissues, the number of interactions increased between cartilage and SynoFP, and meniscus and synoFP, suggesting active immune signaling during OA. Strong intercellular interactions were observed between cell types (Fig. 3d; Fig. S2d). MSCs, fibroblasts, SMCs, chondrocytes, TSCs, neurons, fibroblasts, and macrophages were the major senders of the signals, whereas MSCs, macrophages, neurons, and TSCs were the major receivers (Fig. 3e). OA promotes intricate processes, such as cartilage degradation, inflammation, and pain, which are influenced by specific signaling pathways^43–48^. The top 10 most active signaling pathways in OA were COLLAGEN, FN1, MIF, LAMININ, APP, MHC-II, CD99, CypA, MHC-I, and CXCL (Fig. 3f). Additional pathways, such as prostaglandins, MK, VISFATIN, ANGPTL, and THBS, were highly active in OA (Fig. 3f; Fig. S2d).

Pseudotime trajectory analysis identified the terminally differentiated chondrocytes increased in cartilage as well as meniscus since both these tissues contributed to the highest Pseudotime clusters 1 and 3 (Fig. 3g–i; Fig. S2e). Thus, the reduced TSCs population in the meniscus and synovium OA compared to the healthy knee joint shows a transition to terminally differentiated chondrocytes, which contributes to fibrosis or osteogenic transition in OA.

### MMP13 producing detrimental chondrocytes and synovial fibroblasts were expanded in OA

**Figure 4:**
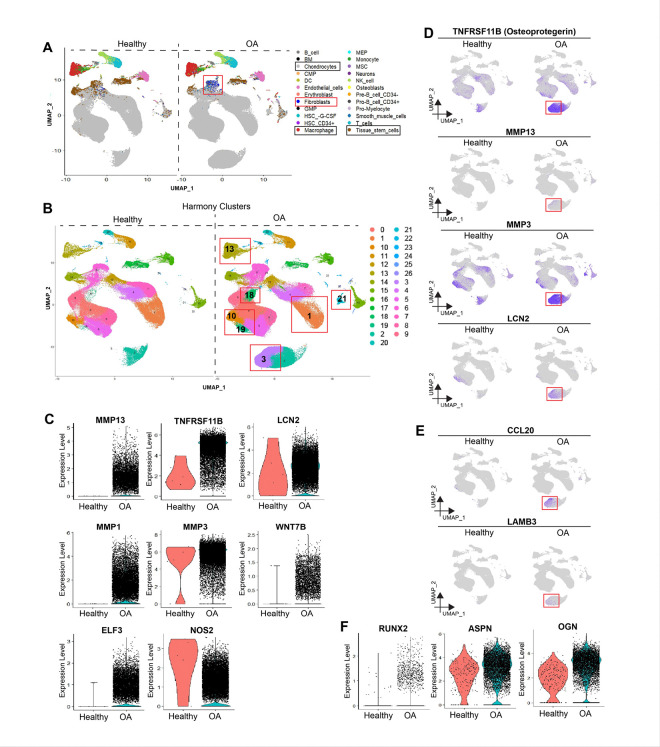
Integration of healthy and OA samples identified an OA-specific degenerative chondrocyte cluster. A) UMAP representation of cell identity in Harmony integrated healthy and OA samples; rectangles show predominant cell types. B) Total cell clusters (n=27) across healthy and OA samples at 0.5 resolution; red boxes highlight the clusters variably present in OA samples. C) Feature plots showing the total expression of top markers of Cluster 3 (MDCs) in both healthy and OA samples; red boxes highlight their expression levels (blue) in Cluster 3. D) Violine plots showing upregulation of chondrocytes expressing OA-specific genes. E) Feature plots showing the expression of CCL20 and LAMB3 in Cluster 3 (MDCs) of OA samples; red boxes highlight their expression levels (blue). F) Violine plots showing chondrocytes expressing mineralization and osteogenic transition-related genes in Cluster 18.

To detect unique cellular and molecular OA-specific variations, healthy and OA knee scRNA-seq samples were integrated and subjected to harmony integration following standard Seurat processing (Fig. S3a). Cell type detection using singleR revealed variability in the population of major cell types, such as chondrocytes, TSCs, macrophages, and most importantly, expansion of synovial fibroblasts in OA (Fig. 4a, Fig. S3b, c). To correlate with the individual analyses described above, a stacked bar plot was generated and compared the cell proportion in both the sample types (Fig. S3d). Clustering was performed at a resolution of 0.5 and generated nearly 27 cell clusters with clusters 3, 10, 18, 19, and 21 expanded, whereas major cluster 1 was depleted in the OA samples (Fig. 4b). All marker analyses were performed to determine the molecular identity of these clusters (Table S1).

OA-depleted Cluster 1 expressed genes, such as CYTL1, CLEC3A, S100B, and FRZB, which are effector chondrocyte (EC) markers^49^ (Table S1). These EC also produce WISP3^50^, CTGF^51^, CYR61^51^, GREM1^52^, and WIF1^53^, which play roles in chondrocyte protection, angiogenesis, and ECM remodeling (Table S3).

The OA-specific chondrocyte cluster, Cluster 3 (Fig. 4b), which is absent in healthy samples, contains specific Detrimental Chondrocytes (DCs) that promote cartilage/ECM degeneration, chondrocyte-to-osteoblast transition, and inflammation-related factors. DCs were enriched with MMP13^54^, TNFRSF11B (osteoprotegerin)^55^, LCN2^56,57^, MMP1^58^, MMP3^59^, ELF3^60,61^, WNT7B^53^, and NOS2^62^ (Fig. 4c) and are referred to as MMP13 producing DCs (MDCs). Next, we confirmed that MDCs primarily produced OA-specific markers (Fig. 4d; Fig. S3e, f). MDCs specifically produce CCL20 and LAMB3, which are important factors in inflammatory pain and cartilage damage, respectively^63,64^ (Fig. 4e).

Cluster 18 was exclusively present in OA samples and was molecularly aligned with the previously described pathogenic chondrocyte subpopulation owing to the higher expression of POSTN and ZEB1^29^. It highly expressed genes, such as NELL1^65,66^, LRRC15^67^, and COL3A1^68^, which play a role in OA progression via osteogenesis, fibrosis, and ECM degeneration (Table S1). In addition, cluster 18 also expressed RUNX2, ASPN, and OGN, which are known to promote mineralization and osteoblasts (Fig. 4f). Cluster 18 expressing genes showed negligible expression in the MDCs. Thus, cluster 18 and MDCs may contribute to worsening disease pathology in human knee OA.

Cluster 10 showed slightly elevated levels of OA-expressing CHI3L1, CHI3L2, MT1G, MT1H, and MT1E regulatory chondrocyte markers (RegC)^29^. Cluster 19 comprised chondrocytes highly expressing IL10^69^, TM4SF1^70^, CHADL^71^, MIA^72^, MT1G^73^, and COL2A1, which are associated with cartilage protection, matrix–proteoglycan synthesis, and reverse calcification and degeneration. Therefore, clusters 10 and 19 appear to expand because of a compensatory response to protect the knee joint from the degenerative effects of OA.

Overall, MMP13 expressing DCs and cluster 18 expressing factors could play a role in specific pathologies and mechanisms in the development of OA, whereas restoring cluster 1 and 19 factors could reverse OA. In addition, expansion of the fibroblast population may result in fibrosis in the synovial region, adding a layer to the pathophysiology of OA.^74^

Synovium and meniscus are the primary residences for TSCs that are depleted in OA.

**Figure 5:**
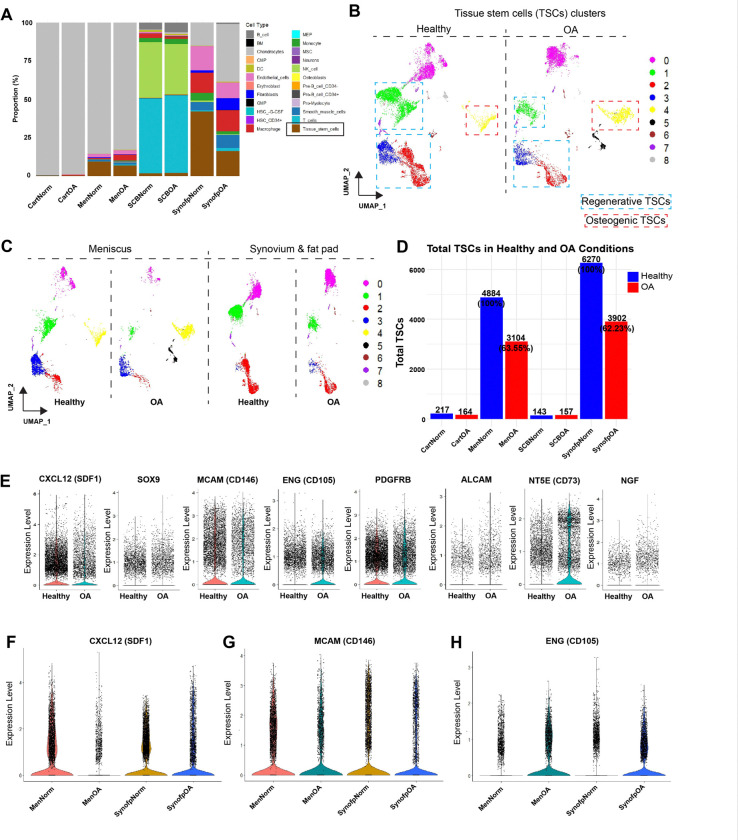
Synovium and meniscus are primary residences for tissue stem cells depleted due to OA. A) Stacked bar plots show comparative cell type proportion in each tissue type during healthy and OA conditions; TSCs are shown in brown color and highlighted in a rectangle. B) UMAP shows the drastic depletion of the TSCs pool in OA compared to the healthy samples. C) Extraction and UMAP visualization of TSCs pool in meniscus and SynoFP during healthy and OA conditions. D) Bar plots show levels of total TSCs in all four tissue types: CartNorm: Healthy cartilage, CartOA: OA cartilage, MenNorm: Healthy meniscus, MenOA: OA meniscus, SCBNorm: Healthy subchondral bone, SCBOA: OA subchondral bone, SynofpNorm: Healthy synovium & infrapatellar fat pad, SynofpOA: OA synovium & infrapatellar fat pad; (Blue: Healthy samples, Red: OA samples). E) Violine plots show TSCs expressing chondroprogenitor and stem cell markers in healthy and OA conditions. F-H) CXCL12 (SDF1), MCAM (CD146), and ENG (CD105, Endoglin) expressing TSCs from meniscus and SynoFP in healthy and OA conditions.

The TSC population was significantly reduced in the SynoFP and menisci. In the meniscus, the TSCs proportion decreased from ~ 9.5% to ~ 6.8%, and SynoFP from ~ 42.2% to ~ 16.4%, which can also be correlated with the expansion of the differentiated chondrocyte population in SynoFP (from ~ 15% to ~ 37.5%) during OA (Fig. 5a). For further analysis, we extracted TSCs from the total cell clusters and generated UMAP to visualize reduced regenerative (Chondrogenic factors producing) and increased osteogenic TSCs in OA (Fig. 5b). As TSCs primarily reside in the meniscus and SynoFP, we generated UMAP to observe the different TSCs contributing to the different TSC subtypes (Fig. 5c). TSCs decreased by 36.45% in the meniscus and 37.77% in SynoFP compared with their respective healthy controls (Fig. 5d). We observed a reduction in the number of regenerative TSCs that produced CXCL12 (SDF1), SOX9, ACAN, MCAM (CD146), PDGFRB, BMPR1B, ENG (CD105/Endoglin), and NGFR (Fig. 5e, Fig. S4a, b). Previously identified chondroprogenitor and stem cell markers that express TSCs have also been affected^10–12^. These TSCs were SDF1 + ^12^, CD146 + ^75^, CD105 + ^76,77^,^77,78^ (Fig. 5f–h).

Interestingly, osteogenic TSCs expressing ALCAM, NT5E (CD73), NGF, and BDNF were increased in OA. NT5E (CD73) is known to increase in OA (Fig. 5e) ^79–81^. Comparative analysis of knee joint tissue compartments identified that the meniscus and synovium are the primary residences of tissue stem cells, which are significantly depleted during OA, contributing to the worst pathological outcome.

Elevated levels of unique IL1B-IL6-NOS2-TNF and pain markers P2RX7-producing macrophages contribute to OA pathophysiology.

**Figure 6:**
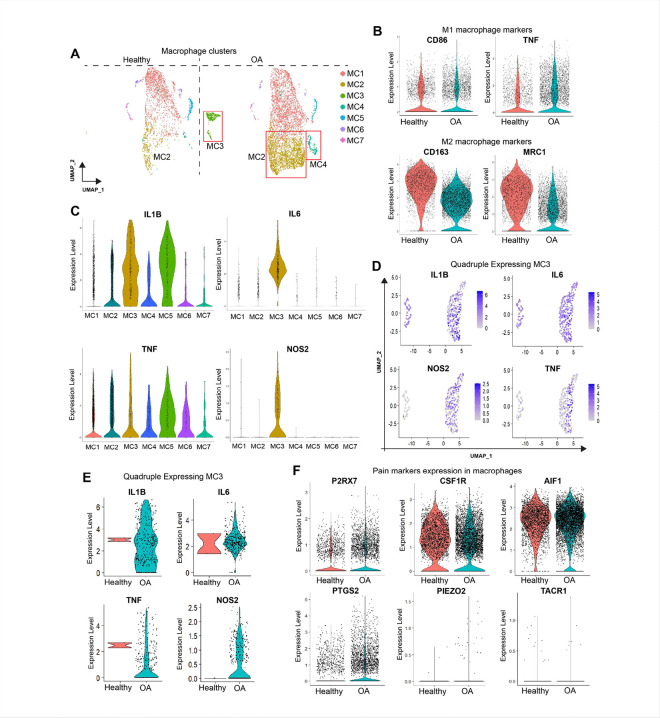
Increased levels of novel quadruple positive inflammation and pain-associated macrophages in OA. A) Extraction and UMAP visualization of the total macrophages in healthy and OA samples. Red boxes highlight the macrophage subtypes enriched in OA samples: MC2, MC3, and MC4 (MC: Macrophage Cluster). B) Visualization of M1 and M2 macrophages in healthy and OA samples, where we used CD86 and TNF as classical M1 macrophage markers and CD163 and MRC1 as classical M2 macrophage markers. C) Violine plots showing significant inflammatory markers expressing MCs, where MC3 stands out as a unique cluster expressing all four inflammatory markers (IL1B, IL6, TNF, and NOS2) (quadruple markers). D-E) Feature plots and violin plots show MC3 expressing quadruple markers. F) Violine plots showing higher enrichment of macrophages expressing classical pain markers in knee OA.

We hypothesized that OA promotes the infiltration of proinflammatory cells, which initiate a vicious cycle of cytokine production, leading to knee joint inflammation and pain. Integration of healthy and OA samples revealed an expansion of the macrophage population in cluster 13 in OA samples (Fig. 4a, b). Within this macrophage population, we identified seven different subpopulations of macrophages at a resolution of 0.5 and labeled them as macrophage clusters MC1-MC7 (Fig. 6a). MC1 and MC2 were the largest clusters, with MC2 being largely expanded during OA, whereas MC3 and MC4 were exclusively present in OA samples (Fig. 6a).

To determine the levels of pro- and anti-inflammatory macrophages under healthy and OA conditions, we analyzed the levels of proinflammatory M1 (CD86 and TNF) and anti-inflammatory M2 (CD163 and MRC1)-type macrophages. We found that CD86 and TNF-producing macrophages were expanded in OA samples, whereas CD163 and MRC1-expressing macrophages were significantly depleted (Fig. 6b). Feature plot analysis revealed that MC1 mostly expressed M2 macrophage markers, whereas MC2 and MC3 expressed M1 macrophage markers (Fig. S5a, b). Next, we analyzed the expression of the proinflammatory OA-causing markers IL1B, IL6, TNF, and NOS2 within each macrophage cluster (Fig. 6c). Interestingly, IL1B and TNF were highly expressed in OA-specific macrophages, MC2 and MC1, suggesting a novel IL1B + TNF + inflammatory macrophage population, irrespective of their M1 and M2 status. These IL1B + and TNF + macrophages may serve as novel prognostic or diagnostic biomarkers of OA pathophysiology.

Notably, we discovered that MC3, a unique OA-specific macrophage cluster, is the only cluster that expresses all four proinflammatory genes: IL1B, NOS2, IL6, and TNF (Fig. 6c, Fig. S5a, e). Next, we extracted MC3 from the total macrophage clusters and confirmed the significantly upregulated expression of these genes in OA (Fig. 6d, e). We termed this novel macrophage subpopulation quadruple-positive (IL1B + IL6 + NOS2 + TNF+) inflammatory macrophages that worsen the disease outcome in terms of joint inflammation and pain in patients with OA.

Finally, we analyzed the expression of classic pain marker genes in total macrophages to understand the association between OA macrophages and joint pain. As expected, P2RX7, CSF1R, AIF1, and PTGS2 (COX2)-producing macrophages were highly expanded in OA samples (Fig. 6f). However, two well-studied OA pain markers, PIEZO2 and TACR1, are not expressed in macrophages. Thus, we conclude that clinical knee OA inflammation and pain arise due to the accumulation of unique dual- and quadruple-positive proinflammatory macrophages, which need to be functionally characterized in a large cohort of clinical samples in the future.

## Discussion

An atlas was constructed by integrating single-cell RNA sequencing profiles of the cartilage, meniscus, synovium, and subchondral bone, which showed specific differences in health and OA knee. The analysis of the molecular landscapes of healthy and OA knees found that OA samples have increased cell-cell communications across the tissues, suggesting active TSCs and immune signaling during OA. However, terminally differentiated chondrocytes in OA originated from the Tissue stem cells (TSCs) residing in the synovium and meniscus identified through pseudotime trajectory analysis. Our findings highlight the synovium as a hotspot of degenerative and regenerative changes. TSCs, cross-tissue interaction, and inflammation affect cartilage integrity and indicate OA-specific alterations initiated in synovial tissue.

As mentioned, TSCs primarily reside in the synovium. Regenerative TSCs were depleted in OA. This dynamic change reduces regeneration and increases degeneration. Regenerative TSCs^10–12^ express markers such as CXCL12 (SDF1), SOX9, ACAN, MCAM (CD146), PDGFRB, BMPR1B, ENG (CD105/Endoglin), and NGFR. SDF1-expressing TSCs are explicitly depleted in the meniscus, and studies have shown that SDF1 is required for chondroprogenitor cell migration during post-injury tissue repair in the meniscus^12^. MCAM+ (CD146+) stem cells have previously shown a more significant migration potential towards degenerated intervertebral discs^75^. CD105 promotes chondrogenesis of synovium-derived MSCs^76,77^. Osteogenic TSCs, which express ALCAM, NT5E (CD73), NGF, and BDNF, were expanded in OA. ALCAM + cells can differentiate into osteoblasts, adipocytes, chondrocytes, and stromal cells, which support osteoclastogenesis, hematopoiesis, and angiogenesis^82^. NT5E is a marker of resting chondrocytes^81^ and is upregulated in OA^79–81^. Expanding fibroblasts in synovium may contribute to fibrosis in OA knees. There was a substantial elevation in inflammation- and pain-related macrophages in OA, including unique macrophage subpopulations with dual positive (IL1B and TNF) and quadruple positive (IL1B, TNF, IL6, and NOS2) nature.

OA-specific MDCs were enriched with degradative factors such as LCN2^56^, TNFRSF11B^55^, MMP1, MMP3, MMP13, NOS2, WNT7B^53^, and ELF3^60^,^61^, which play significant roles in extracellular matrix destruction, bone remodeling, and cartilage degradation. OA-specific cluster 18 is enriched with RUNX2^66^, ASPN, and OGN, which are markers of mineralization and osteoblast characteristics^55^ that contribute to stiffness. OA-specific MDCs were enriched in CCL20 and other markers associated with inflammation and pain. In the presence of CCL20, cartilage released more MMP-1, MMP-13, PGE2, GAG fragments, and IL-6, whereas collagen type II mRNA expression was inhibited^63^.

The limitation of this study is that, although we generated a unique scRNA-seq atlas, the inclusion of a large number of samples with various stages of OA will provide the detailed mechanism for OA progression. OA disproportionately affects underserved populations^83,84^ and females. The detailed temporal and spatial appearance of RNA from the time of an inciting injury through the initiation and progression of OA pathophysiology, its proteomics profile, and actual tissue changes need to be performed. Future studies should involve well-controlled clinical cohorts with age- and sex-matched large cohorts of samples and standardized tissue extraction procedures.

In conclusion, the synovium and meniscus are the most damaged compartments, and synovium regeneration could have potential therapeutic opportunities considering highly dysregulated homeostasis. Atala et al. showed that the synovial environment causes cartilage deterioration and regeneration^85^. OA pain may be a consequence of resident TSCs depletion and proinflammatory changes in the synovium and meniscus. Moreover, dual- and quadruple-positive macrophages could serve as prognostic markers to identify OA severity pre-and post-treatment. As there are no FDA-approved drugs, our study shows that future clinical trials should consider the involvement of synovium and meniscus regeneration when designing future OA disease-modifying drugs. Our analysis also emphasizes the heterogeneity within the meniscus and bone compartments, revealing distinct cellular niches that may influence disease outcomes. Understanding the role of these niches in OA pathogenesis could lead to more targeted and effective treatments. This atlas provides a valuable resource for the scientific community, offering new perspectives on cellular and molecular mechanisms and opening up exciting possibilities for developing targeted therapies aimed at halting or reversing disease progression. Future studies should build on these findings to further elucidate the intricate cellular dynamics within the OA joint and translate these insights into clinical applications.

## Data Availability

The human knee OA single-cell RNA-seq data used in this study are publicly available under the following accession ID: cartilage (GSE220243, GSE169454, and GSE255460); meniscus (GSE220243); synovium and IPFP (GSE216651); and subchondral bone (GSE196678).
